# The treatment of autism with low-dose phenytoin: a case report

**DOI:** 10.1186/1752-1947-9-8

**Published:** 2015-01-16

**Authors:** Philip D Bird

**Affiliations:** PO Box 680, Maroochydore, 4558 QLD Australia

**Keywords:** Autism, Low dose phenytoin, Treatment, Social cognition

## Abstract

**Introduction:**

The drug treatment of autism spectrum disorders is often poorly tolerated and has traditionally targeted associated conditions (such as inattention or irritability) that frequently coexist, with limited benefit for the core social deficits. Here, I describe the novel use of a low dose of the anti-epileptic phenytoin to enhance social functioning in a patient with an autism spectrum disorder.

**Case presentation:**

I present the case of a 19-year-old Caucasian man with autism spectrum disorder treated with stimulant medication since early childhood. He experienced long-standing difficulties in establishing and maintaining relationships and reading social cues, and was socially isolated. Within 10 minutes of a single sublingual low dose of phenytoin there was an immediate observable improvement in his eye contact and integration of both verbal and non-verbal communication. This enhanced social functioning associated with his adherence to the low-dose phenytoin therapy was maintained for over 18 months of follow-up. These clinical observations were supported by ratings using the Autism-Spectrum Quotient and the Depression, Anxiety and Stress Scales, recorded pre-treatment and after seven months on 5mg phenytoin.

**Conclusion:**

This case report provides the first potential evidence that a low dose of phenytoin, a widely used and well tolerated anti-epileptic medication, may be capable of modifying the core social cognitive deficits associated with autism spectrum disorders. While acknowledging this is a single case study, the lack of availability of safe and effective treatments to address the important core deficits associated with autism spectrum disorders makes this case noteworthy.

## Introduction

Autism is a neurodevelopmental disorder characterized by dysfunction in three core behavioral dimensions: repetitive behaviors, social deficits and language abnormalities. Autism is assigned to a spectrum of disorders that are referred to as autism spectrum disorders (ASDs), distinguishable in the severity of symptoms [1]. Autism is the model disorder of social dysfunction - a key inability to respond appropriately to social cues, including failure to accurately interpret facial expressions [2]. Additional social deficits include unusual eye contact, limitations in facial expression directed to other people, atypical social engagement and responsiveness, difficulty with peer relationships, lack of awareness of other people’s thoughts and feelings, poor communication skills, and difficulty initiating social contacts through verbal or non-verbal means [3].

Only two medications, risperidone and aripiprazole, have so far been approved by the United States Food and Drug Administration for the treatment of autism, and these are for the associated behavioral disturbance [4]. Although effective treatments, they are associated with side-effects including sedation, cognitive impairment, weight gain and metabolic disturbance [5].

I observed that patients with attention deficit hyperactivity disorder (ADHD) who on their stimulant therapy often still experienced persistent affective instability. The addition of sodium valproate was undertaken in an attempt to improve these symptoms. Unexpectedly, a number of patients reported an improvement in their ability to maintain eye contact during conversations, which in turn enabled them to better read non-verbal social cues and enhanced their comprehension and enjoyment of social interaction. Significantly, this beneficial effect of sodium valproate appeared to have a narrow therapeutic window, with the optimal range between 50 and 200mg daily. A complete loss of efficacy frequently occurred above a dose of 400mg. Similarly, phenytoin is an anti-epileptic that has shown to have some benefit in the treatment of bipolar mood disorder [6].

I present a case of a man with a childhood diagnosis of ASD and co-morbid ADHD with persistent disruptive behavioral difficulties despite long-term treatment with stimulant medication. Following the addition of a very low dose of phenytoin there was an improvement in multiple areas of his functioning, in particular in the core areas of social interaction impaired in ASDs. He was for the first time able to sustain eye contact, demonstrated more spontaneous facial expression, and began to use gestures during conversation. He also appeared to enjoy and benefit from the company of others.

Evaluations of the effects of medication pre- and post-treatment with phenytoin were two-fold: behavioral observations (for example, client and carer reports, clinician’s observations) and formal ratings on behavioral scales (The Depression Anxiety and Stress Scales (DASS) and The Autism-Spectrum Quotient (AQ)).

The DASS is a 42-item self-administered questionnaire designed to measure the magnitude of three negative emotional states: depression, anxiety and stress [7]. This instrument is considered to be an appropriate and reliable measure of overall improvement that may correlate with overall functioning. Depression and anxiety are among the most common ASD co-morbidities [8] and correlate to significantly poorer life functioning [9].

The AQ, although not a diagnostic instrument, is useful in identifying the extent of autistic traits shown by an adult of normal intelligence. It was designed as a screening tool but also provides a quantitative approach to the measurement of ASD symptoms and is considered to have established test-retest reliability [10]. It was therefore useful in the assessment of clinical improvement.

## Case presentation

My patient was a 19-year-old man diagnosed in early childhood with ADHD and ASD. He was referred by his local doctor for ongoing treatment as an adult. He had experienced long-term difficulties in establishing and maintaining lasting relationships and reading social cues. His conversations were awkward and restricted to a narrow range of subjects and he was frequently impulsive and insensitive during these interactions. These impairments had contributed to his increasing social isolation and conflict within the family environment. He had a low threshold of irritation and intolerance of noise, light and crowds. There was also a long history of outbursts of anger, physical intimidation and frequent destruction to property at home. He was also disorganized, forgetful and had difficulty completing tasks. He has always been overactive, restless and impulsive. There was no history of pervasive mood disturbance.

### Developmental and social history

There were early concerns about a potential developmental delay and he was referred for a pediatric assessment at age two years. He received speech therapy prior to school and also repeated his pre-school year. Although he was of average intellect, his significant and specific learning, behavioral and communication difficulties warranted a placement in a special education unit to manage his needs. He experienced difficulties in both reading and writing and he performed below all national standards of literacy and numeracy. Reading remained effortful and unrewarding, with difficulties focusing on and reading along a line of text. At the age of seven he had an occupational therapy assessment, which reported difficulties with coordination, fine motor control, planning and sequencing. In the classroom he was reported to be distracted and restless, presented with extreme outbursts of anger and frequently ran away. He had poor interpersonal skills, established few friendships, had odd idiosyncratic language and repetitive hand flapping, and had a history of being bullied.

He commenced stimulant medication during early childhood and had trials of immediate-release dexamphetamine and methylphenidate with improvement in classroom behavior. He had little interest or motivation in taking his medication, resulting in generally poor adherence. Without stimulant medication his behavior quickly deteriorated.

Since leaving school he had obtained a number of part-time unskilled jobs for eight to ten hours per week. He was, however, unable to maintain them because of behavioral and communication difficulties. He did not smoke cigarettes or consume alcohol or illicit substances. There was no significant family psychiatric history.

On presentation, he was taking methylphenidate extended-release 72mg daily via an osmotic-controlled release oral delivery system and melatonin 4mg every night. He had been prescribed this medication for the previous two years.

### Clinical assessment

During my clinical assessment, he was constantly restless, appeared disinterested and was easily distracted. He made little eye contact either when listening or speaking and his non-verbal interaction was limited. His answers to questions were minimal and his speech lacked normal prosody. His blood pressure and pulse were within the normal range although he was significantly overweight with a body mass index of 34kg/m^2^ (weight 102kg, height 1.72m).

Following completion of his assessment an additional dose of methylphenidate was recommended in the late afternoon to try and reduce his frequent aggression at this time. However, he stated that this suggestion was of no interest to him and he did not care that others might be upset by his behavior.

During the three months following his initial assessment there was no alteration to his pharmacotherapy and no improvement in his behavior. As a consequence of his persistent social difficulties the option of a trial of low-dose phenytoin was discussed. It was explained to my patient and his mother that improvements in social functioning had been observed by myself and been reported by other patients who also had taken low-dose phenytoin; although, these effects had not been demonstrated outside the clinic nor was this an approved indication for the medication. My patient and his mother signed a disclosure and informed consent document and a trial of medication was organized for the following consultation.

Two weeks later a sublingual test dose of approximately 2mg phenytoin was administered. Prior to this dose my patient’s interaction reflected his usual behavior, with little interest and social engagement. He was reluctant to participate in conversation and his verbal responses were minimal and lacked elaboration; there was only minimal facial expression, which appeared to be unconnected to the content of his speech. Within 10 minutes of taking the sublingual phenytoin he reported a reduction in the effort required to contribute to conversation and was able to sustain eye contact both when listening and speaking. He was surprised about the effortless nature of his eye gaze and also commented that he had never done this before, and that previously he had always found it easier to avoid eye contact when speaking. He was now experiencing the reverse, finding it harder to break away from the mutual eye gaze. He stated he felt more relaxed and was less distracted by other environmental visual and sensory stimuli. His non-verbal interaction, demonstrated by the raising of his eyebrows, smiling and nodding of his head, appeared to be more spontaneous and natural. During this assessment he read aloud two standardized examples of a text, pre- and post-administration of the phenytoin. He reported a reduction in the effort required and improved comprehension and accuracy. He stated that he was no longer as distracted by the other lines of text on the page, which previously had resulted in becoming overwhelmed and lost on the page. His speech was also more fluid with more appropriate intonation. He denied any adverse effects of the test dose. The following day he started taking compounded 2mg phenytoin capsules in the morning in conjunction with his methylphenidate.

After two weeks both he and his mother stated that his communication with the family had improved and there had been no aggressive outbursts. During the consultation he was noticeably more engaged and appeared to enjoy the interaction. His ability to maintain eye contact and his non-verbal communication - facial expression, head movements and posture -were more synchronized and spontaneous.

Over the next four weeks he became inconsistent in taking the phenytoin, and then ceased altogether. His behavior reverted to the previous pattern of poor social interaction; he became oppositional with outbursts of anger and physical violence.

Nine months later he resumed taking the phenytoin, this time as a single 4mg capsule in the morning. After his first dose there was an improvement of his social behavior similar to his previous response, although there was an apparent deterioration in the late afternoon. The dose was increased from 4mg to 5mg and a larger capsule formulated to try and prolong the release of the phenytoin. This appeared to achieve a more consistent improvement in behavior throughout the day, evident both at home and at work. Increases in the dose above 5mg were not associated with any additional benefit. He remained on the 5mg dose of phenytoin for over 18 months and reported that his work performance had consistently improved sufficient to increase his working hours and his level of responsibility. The violence and destruction at home abated. His confidence improved and for the first time he has established and sustained peer-appropriate friendships.

**Figure 1:**
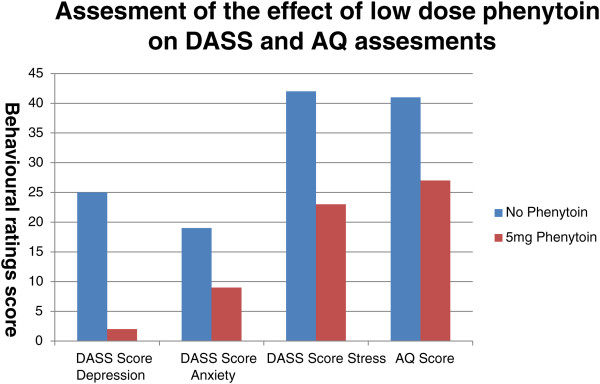
**Graphical presentation of behavioral ratings prior to and after seven months of continuous treatment with 5mg phenytoin.** DASS: Depression Anxiety Stress Scales (depression and anxiety clinically significant cut-off: 12; reliable change: 5 scale points; stress clinically significant cut-off: 14; reliable change: 7 scale points); AQ: Autism-Spectrum Quotient (a score of 32 indicates clinically significant levels of autistic traits).

His behavioral ratings prior to treatment with phenytoin and after seven months on the 5mg phenytoin are presented in Figure 1. The manner in which my patient endorsed the items on the AQ highlighted a reduction of symptoms, with the most robust improvements in his enjoyment of social occasions, ability to cope with chit chat, and his preference to be with people rather than be alone. His total AQ score dropped from 41 pre- to 27 post-treatment with phenytoin; a score of 32 or more is considered to indicate clinically significant levels of autistic traits [10]. Before treatment with phenytoin, his DASS ratings recorded both depression and anxiety as severe and stress as very severe. Post-treatment, there was robust change in all domains, with an absence of depressive symptoms and with both anxiety and stress being recorded as moderate.

## Discussion

ASDs are characterized by social deficits and communication difficulties, and stereotyped or repetitive behaviors and interests. The social deficits have been noted in autism as a failure to understand the intentions and mental states of others [11]. The treatment of ASD has remained difficult. The only psychotropic interventions with research data strong enough to obtain a rating of ‘established evidence’ [4] have been within the antipsychotic class and are for the treatment of the behavioral manifestations of ASDs.

In this case report I have described the clinical presentation of a patient with ASD who benefited clinically with increased social functioning from treatment with low-dose phenytoin. The initial sublingual test dose provided a more rapid onset of therapeutic effect compared with the variable absorption often associated with oral phenytoin [12], although the speed of response was consistent with other pharmaceuticals agents [13]. The medication was well tolerated and resulted in improvements in multiple domains including the core social deficits associated with ASD. Perhaps the most significant social change was my patient’s ability to establish and maintain eye contact during conversation. This provided an enhanced and more comfortable social engagement and a sense of real-time feedback on the outcome of conversation that previously had not occurred. His ability to sustain eye contact and interpret non-verbal communication may have contributed to a reduction in social anxiety and the improvement in quality of life reported by my patient and his family.

This clinical case report provides potential evidence that a low dose of a widely used anti-epileptic medication, phenytoin, may be capable of modifying the core social cognitive deficits associated with ASDs. These clinical improvements were reflected and maintained for over seven months in the AQ and DASS.

My previous clinical observations in similar patients led to use of sub-therapeutic doses of an anti-epileptic medication to improve social cognition. This discovery was unexpected and I am unaware of prior similar scientific reports. The pharmacological action of anti-epileptic medication in ASD is not entirely unexpected because the conditions may have a common neurodevelopment origin, although a precise link between these two pathologies is still undetermined [14]. The ability of a low dose of an anti-epileptic medication to rescue autistic behavior in an animal model has already been established. Dravet’s syndrome (DS) is a form of autism and epilepsy in which there is a *SCN1A* mutation that encodes for VGSC type-1 (NaV1.1) [15], the primary sodium channel in the GABAergic interneurons. An *SCN1A* haplo-insufficiency results in a reduction of GABAergic transmission. Han *et al.* reported that treatment with low-dose (1/40th of the therapeutic dose) clonazepam, a positive allosteric modulator of GABA_A_ receptors, completely rescued the abnormal social behaviors and deficits in fear memory in the mouse model of DS, demonstrating that they are both caused by impaired GABAergic function and sensitive to very low (sub-therapeutic) doses of an anti-epileptic medication [16].

ASDs are increasingly thought to be characterized by a disruption in the excitatory and inhibitory balance of neural activity [17]. Mutations in genes involved in the expression of excitatory and inhibitory neurotransmitters (for example, glutamate and gamma-aminobutyric acid (GABA)) have been identified in individuals with ASD [18]. This has been supported by increasing evidence that autism and related developmental conditions involve a GABA deficit [19]. More recently, strong evidence has been building for a role of lesions in neuronal voltage-gated sodium channel (VGSC) alpha subunits in typical polygenic ASD [20]. These channels are also the therapeutic targets of phenytoin in the brain [15, 21]. The VGSC gene family comprises nine homologous members, *SCN1A* to *SCN11A*, that encode the sodium-selective ion channels NaV1.1 to NaV1.9. *SCN1A*, *SCN2A*, *SCN3A*, SCN7A and *SCN8A* are associated with ASD [20]. Whole-exome sequencing of nearly 1000 individuals identified *SCN2A* as the sole gene in which two independent probands had non-sense variants that disrupted the same gene, a highly significant result [22]. The importance of VGSCs in typical polygenic ASD was also supported by a separate large sequencing study that found *de novo* protein-altering mutations in the gene in probands with ASD [23].

I hypothesize that, in a similar mechanism to the low-dose clonazepam in this animal model of autism, low-dose phenytoin may enhance GABA neurotransmission, thereby correcting the imbalance between the GABAergic and glutaminergic systems.

## Conclusions

While acknowledging this is an anecdotal single case study, the absence of safe and effective treatments to address the core deficits associated with ASD makes the improvements demonstrated with low-dose phenytoin in this case noteworthy. The mechanism of action of low-dose phenytoin is not known. However, I propose a hypothesis to explain the therapeutic action based on our current understanding ASD together with an associated animal model. An independent placebo-controlled proof-of-concept study of low-dose phenytoin will be required to determine whether the clinical improvement demonstrated in this case can be replicated in a larger cohort.

## Consent

Written informed consent was obtained from the patient and his parent for publication of this case report and accompanying data. A copy of the written consent is available for review by the Editor-in-Chief of this journal.

## References

[1] Erdmann J: **Broad collaborations bring new energy to autism therapeutics.** *Chem Biol* 2011, **18:**142–143. 10.1016/j.chembiol.2011.02.006 10.1016/j.chembiol.2011.02.006 21338911

[2] American Psychiatric Association: *Diagnostic and Statistical Manual of Mental Disorders, 4th Ed, Text Revision*. Washington, DC: American Psychiatric Association; 2000.

[3] Tager-Flusberg H: **The origins of social impairments in autism spectrum disorder: studies of infants at risk.** *Neural Netw* 2010, **23:**1072–1076. 10.1016/j.neunet.2010.07.008 10.1016/j.neunet.2010.07.008 PMC2956843 20800990

[4] Siegel M, Beaulieu AA: **Psychotropic medications in children with autism spectrum disorders: a systematic review and synthesis for evidence-based practice.** *J Autism Dev Disord* 2012, **42:**1592–1605. 10.1007/s10803-011-1399-2 10.1007/s10803-011-1399-2 22068820

[5] Newcomer JW: **Second-generation (atypical) antipsychotics and metabolic effects: a comprehensive literature review.** *CNS Drugs* 2005,**19**(Suppl 1)**:**1–93. 10.2165/00023210-200519001-00001 15998156

[6] Bersudsky Y: **Phenytoin: an anti-bipolar anticonvulsant?** *Int J Neuropsychopharmacol* 2006, **9:**479–484. 10.1017/S1461145705006140 10.1017/S1461145705006140 16202184

[7] Parkitny L, McAuley J: **The Depression Anxiety Stress Scale (DASS).** *J Physiother* 2010, **56:**204. 10.1016/S1836-9553(10)70030-8 10.1016/s1836-9553(10)70030-8 20795931

[8] Simonoff E, Pickles A, Charman T, Chandler S, Loucas T, Baird G: **Psychiatric disorders in children with autism spectrum disorders: prevalence, comorbidity, and associated factors in a population-derived sample.** *J Am Acad Child Adolesc Psychiatry* 2008, **47:**921–929. 10.1097/CHI.0b013e318179964f 10.1097/CHI.0b013e318179964f 18645422

[9] Mattila M-L, Hurtig T, Haapsamo H, Jussila K, Kuusikko-Gauffin S, Kielinen M, Linna S-L, Ebeling H, Bloigu R, Joskitt L, Pauls DL, Moilanen I: **Comorbid psychiatric disorders associated with Asperger syndrome/high-functioning autism: a community- and clinic-based study.** *J Autism Dev Disord* 2010, **40:**1080–1093. 10.1007/s10803-010-0958-2 10.1007/s10803-010-0958-2 20177765

[10] Baron-Cohen S, Wheelwright S, Skinner R, Martin J, Clubley E: **The autism-spectrum quotient (AQ): evidence from Asperger syndrome/high-functioning autism, males and females, scientists and mathematicians.** *J Autism Dev Disord* 2001, **31:**5–17. 10.1023/A:1005653411471 10.1023/a:1005653411471 11439754

[11] Baron-Cohen S, Wheelwright S, Hill J, Raste Y, Plumb I: **The “Reading the Mind in the Eyes” test revised version: a study with normal adults, and adults with Asperger syndrome or high-functioning autism.** *J Child Psychol Psychiatry* 2001, **42:**241–251. 10.1111/1469-7610.00715 11280420

[12] Gibberd FB, Webley M: **Studies in man of phenytoin absorption and its implications.** *J Neurol Neurosurg Psychiatry* 1975, **38:**219–224. 10.1136/jnnp.38.3.219 10.1136/jnnp.38.3.219 PMC491899 1151402

[13] Bartlett JA, van der Voort MK: **Understanding the oral mucosal absorption and resulting clinical pharmacokinetics of asenapine.** *AAPS PharmSciTech* 2012, **13:**1110–1115. 10.1208/s12249-012-9839-7 10.1208/s12249-012-9839-7 PMC3513449 22936407

[14] Genovesi S, Provenzano G, Dunleavy M, Sgadò P, Bozzi Y: **GABAergic dysfunction in autism and epilepsy, autism - a neurodevelopmental journey from genes to behaviour.** In *A Neurodevelopmental Journey from Genes to Behaviour*. Edited by: Rijeka VE, Valsamma E. Rijeka: InTech; 2011:25–48.

[15] Lipkind GM, Fozzard HA: **Molecular model of anticonvulsant drug binding to the voltage-gated sodium channel inner pore.** *Mol Pharmacol* 2010, **78:**631–638. 10.1124/mol.110.064683 10.1124/mol.110.064683 PMC2981395 20643904

[16] Han S, Tai C, Westenbroek RE, Yu FH, Cheah CS, Potter GB, Rubenstein JL, Scheuer T, de la Iglesia HO, Catterall WA: **Autistic-like behaviour in Scn1a+/− mice and rescue by enhanced GABA-mediated neurotransmission.** *Nature* 2012, **489:**385–390. 10.1038/nature11356 10.1038/nature11356 PMC3448848 22914087

[17] Cornew L, Roberts TPL, Blaskey L, Edgar JC: **Resting-state oscillatory activity in autism spectrum disorders.** *J Autism Dev Disord* 2012, **42:**1884–1894. 10.1007/s10803-011-1431-6 10.1007/s10803-011-1431-6 PMC3638261 22207057

[18] Collins AL, Ma D, Whitehead PL, Martin ER, Wright HH, Abramson RK, Hussman JP, Haines JL, Cuccaro ML, Gilbert JR, Pericak-Vance MA: **Investigation of autism and GABA receptor subunit genes in multiple ethnic groups.** *Neurogenetics* 2006, **7:**167–174. 10.1007/s10048-006-0045-1 10.1007/s10048-006-0045-1 PMC1513515 16770606

[19] Coghlan S, Horder J, Inkster B, Mendez MA, Murphy DG, Nutt DJ: **GABA system dysfunction in autism and related disorders: from synapse to symptoms.** *Neurosci Biobehav Rev* 2012, **36:**2044–2055. 10.1016/j.neubiorev.2012.07.005 10.1016/j.neubiorev.2012.07.005 PMC4477717 22841562

[20] Schmunk G, Gargus JJ: **Channelopathy pathogenesis in autism spectrum disorders.** *Front Genet* 2013, **4:**222. 10.3389/fgene.2013.00222 PMC3817418 24204377

[21] Catterall WA, Kalume F, Oakley JC: **NaV1.1 channels and epilepsy.** *J Physiol* 2010,**588**(Pt 11)**:**1849–1859. 10.1113/jphysiol.2010.187484 PMC2901973 20194124

[22] Sanders SJ, Murtha MT, Gupta AR, Murdoch JD, Raubeson MJ, Willsey AJ, Ercan-Sencicek AG, DiLullo NM, Parikshak NN, Stein JL, Walker MF, Ober GT, Teran NA, Song Y, El-Fishawy P, Murtha RC, Choi M, Overton JD, Bjornson RD, Carriero NJ, Meyer KA, Bilguvar K, Mane SM, Šestan N, Lifton RP, Günel M, Roeder K, Geschwind DH, Devlin B, State MW: **De novo mutations revealed by whole-exome sequencing are strongly associated with autism.** *Nature* 2012, **485:**1–6. 10.1038/nature10945 PMC3667984 22495306

[23] O’Roak BJ, Vives L, Fu W, Egertson JD, Stanaway IB, Phelps IG, Carvill G, Kumar A, Lee C, Ankenman K, Munson J, Hiatt JB, Turner EH, Levy R, O’Day DR, Krumm N, Coe BP, Martin BK, Borenstein E, Nickerson DA, Mefford HC, Doherty D, Akey JM, Bernier R, Eichler EE, Shendure J: **Multiplex targeted sequencing identifies recurrently mutated genes in autism spectrum disorders.** *Science* 2012, **338:**1619–1622. 10.1126/science.1227764 10.1126/science.1227764 PMC3528801 23160955

